# Protein intrinsic disorder toolbox for comparative analysis of viral proteins

**DOI:** 10.1186/1471-2164-9-S2-S4

**Published:** 2008-09-16

**Authors:** Gerard Kian-Meng Goh, A Keith Dunker, Vladimir N Uversky

**Affiliations:** 1 Center for Computational Biology and Bioinformatics, Indiana University School of Medicine, Indianapolis, IN 46202, USA; 2 Institute for Intrinsically Disordered Protein Research, Indiana University School of Medicine, Indianapolis, Indiana 46202, USA; 3 Institute for Biological Instrumentation, Russian Academy of Sciences, 142290 Pushchino, Moscow Region, Russia

## Abstract

To examine the usefulness of protein disorder predictions as a tool for the comparative analysis of viral proteins, a relational database has been constructed. The database includes proteins from influenza A and HIV-related viruses. Annotations include viral protein sequence, disorder prediction, structure, and function. Location of each protein within a virion, if known, is also denoted. Our analysis reveals a clear relationship between proximity to the RNA core and the percentage of predicted disordered residues for a set of influenza A virus proteins.

Neuraminidases (NA) and hemagglutinin (HA) of major influenza A pandemics tend to pair in such a way that both proteins tend to be either ordered-ordered or disordered-disordered by prediction. This may be the result of these proteins evolving from being lipid-associated. High abundance of intrinsic disorder in envelope and matrix proteins from HIV-related viruses likely represents a mechanism where HIV virions can escape immune response despite the availability of antibodies for the HIV-related proteins. This exercise provides an example showing how the combined use of intrinsic disorder predictions and relational databases provides an improved understanding of the functional and structural behaviour of viral proteins.

## Background

### Goals and objectives

Structures and functions of a large number of viral proteins are not yet totally understood [1-5]. This may account for the continuous need for the development of novel computational and experimental tools suitable for the viral protein analysis. Although experimental techniques remain the major providers of structural and functional knowledge, often, the experiments are expensive or difficult to the point of infeasibility. The use of various bioinformatics tools to predict structure and function represents an alternative approach that is gaining significant attention. Comparative computational studies have opened a new way for easier benchmarking and functional analysis of proteins. Here we examine the usefulness of intrinsic disorder predictions for studying the viral proteins. To this end, a set of biocomputing tools that include relational database design and utilization of disorder prediction algorithms was elaborated.

### Viral protein functions by proteins, location and virus type

Two families of RNA viruses, the *Lentivirinae *(HIV) and the *Orthomyxoviridae *(Influenza), were used in this comparative study. These viral families were selected because they are widely studied due to their involvement in major outbreaks during the last century [5,6]. The Lentiviruses include the HIV and the SIV viruses among others [7], whereas the orthomyxoviruses encompass mainly the various influenza viruses [8].

**Figure 1:**
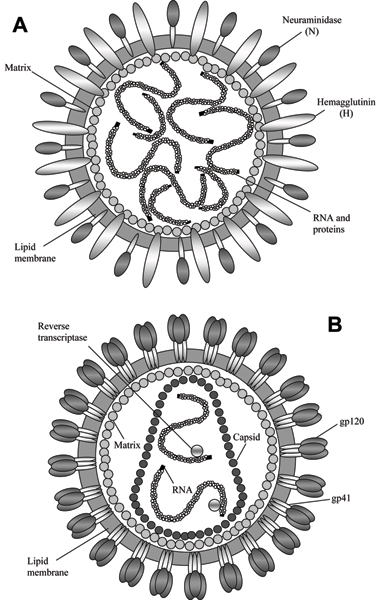
Model structures of the influenza A (A) and HIV-1 (B) virions.

The influenza A virion (which is a complete virus particle with its RNA core and protein coat) is a globular particle sheathed in a lipid bilayer derived from the plasma membrane of its host (Figure 1A). Two integral membrane proteins, hemagglutinin (HA) and neuraminidase (NA), are studded in the lipid bilayer. Beneath the envelope, the matrix formed by matrix proteins M1 and M2 is located. This matrix encompasses eight pieces of the genomic RNA, each in association with many copies of a nucleoprotein (NP), some "non-structural" proteins with various functions (e.g., NS1 and NS2) and several molecules of the three subunits of its RNA polymerase. Sixteen HA subtypes (or serotypes) and nine NA subtypes of influenza A virus have been identified in different virus isolations so far.

HIV is also an enveloped virus. Figure 1B represents a model of its virion. The surface of the HIV virion is the viral envelope made of the cellular membrane, which is acquired when the virus leaves the host cell. Protruding from the envelope is the viral glycoprotein, gp160, which is made up of two component parts, the structural unit (SU), gp120, and the transmembrane (TM), gp41. These two surface proteins play important roles in attachment and penetration of HIV into target cells. Inside the lipid envelope, there is a matrix formed by Gag protein p17, which holds the RNA-containing core in place. This cylindrical core is a proteinaceous capsid made of p24 protein. The capsid contains two copies of the single-stranded RNA genome and three key enzymes: protease, PR (p11); integrase, IN (p32); and reverse transcriptase RT (p66), as well as some other proteins.

Table 1 represents a list of some of the most important proteins analyzed in this study. These proteins are arranged by their approximate location in the HIV and Influenza A virions [7-10]; i.e., according to their proximity to the core where the RNA is housed. The proteins that are located closer to the core are likelier to be involved in interaction with the viral RNA. Note: the exact locations of some of the proteins within the virions are not known as of yet.

**Table 1:** HIV and influenza viral proteins. Some of the most important HIV and influenza viral proteins are listed with a brief description of their functions. They are listed by the order of their location in the corresponding virions [7-10].

Major HIV proteins		
Protein	Function of the Protein	Location

SU(gp120)	Binding of host's CD4 to itself	Virion Surface(SU)
TM (gp41)	Involved in fusion with host	Transmembrane (TM) at the Virion Envelope
MA (p17)	Matrix	Beneath the Enevelope
Vpu^a^	Virion release	
Vpr	Nucleus transportation of viral proteins	Within capsid and beneath matrix
CA (p24)	Main core protein	Nucleocapsid
PR (p11)	Protease.	Within the Core
RT (p66)	Reverse Transcriptase	Within th Core
IN (p32)	Integrase.	Within the Core
Tat^a^	Transcription factor	NA^a^

Major influenza A virus proteins		

Protein	Function/Description	Location

HA	Hemagglutinin allows the attachhment of host's CD4 to itself	Envelope
NA	Neuraminidase cleaves sialic acud group to allow virion release into the extracellular region.	Transmembrane (TM) at the Virion Envelope
MA (M1, M2)	Protein assembly with membrane binding and disassociation	Matrix: Beneath the Enevelope
NS1	Non-Structural Protein. Inhibits RNA splicing. RNA binding	Within capsid and beneath matrix
NS2	Transportation of RNP^b ^to cytoplasm	
NP	Nucleoprotein	Nucleocapsid
PB1	Main core protein	Binds to Nucleocapsid

^a ^Positions of VPU and Tat in the virion is unclear

^b ^Ribonucleoprotein (RNP)

Table 1 shows that proteins similarly located within the virions of different viral types possess significant functional similarities [11]. For example, similar functions can be seen in the surface proteins (gp120, HA, NA) in both influenza A and HIV viruses. Although Table 1 lists major functions for several proteins, it is important to remember that some of the functions are not totally understood or are not known at all [1]. Multi-functionality of a protein is, of course, also possible.

### Intrinsic disorder

Many proteins are intrinsically disordered; i.e., they lack rigid 3-D structure under physiological conditions *in vitro*, existing instead as dynamic ensembles of interconverting structures. Intrinsically disordered proteins [12] are also known by several other names including "intrinsically unstructured" [13] and "natively unfolded." [14-16] While the function of a given protein is often determined by its unique structure, comparative studies on several exceptions to the structure-to-function mechanism led to the realizations that intrinsically disordered proteins share many sequence characteristics and so comprise a distinct cohort. These intrinsically unstructured proteins and regions differ from structured globular proteins and domains with regard to many attributes, including amino acid composition, sequence complexity, hydrophobicity, charge, flexibility [12,15], and type and rate of amino acid substitutions over evolutionary time [17]. Many of these differences between ordered and intrinsically disordered proteins were utilized to develop numerous disorder predictors. The disorder predictors used in this paper are PONDR^®^s (Predictors of Naturally Disordered Regions) VLXT and VL3 [18-21]. We utilized these predictors to address the following question: Can disorder prediction be used to determine or map at least some the functions for viral proteins?

## Results

### Predicted intrinsic disorder in various viruses

Table 2 lists the average percentages of predicted disordered residues (the percentage disorder rate) that have been found in proteins studied by NMR or X-Ray crystallography. They are also divided into PDB-Select 90 proteins [22,23], lentivirus, and influenza A virus. Table 2 shows that the percentage of residues predicted to be disordered by PONDR^® ^VLXT in proteins from a PDB-Select 90 set is 24 ± 2, whereas the corresponding value for PONDR^® ^VL3 predictions is 14 ± 2. Table 2 also shows that predicted disorder is a bit more abundant in lentivirus proteins in comparison with proteins from the influenza virus. The values given in this table are the average percentages of disordered residues in a given dataset, not the average percentages of disordered residues *in each chain*. The former provides a better gauge of the mean since the number of influenza and HIV-related proteins available in PDB [22] is relatively small.

**Table 2:** Abundance of intrinsic disorder in various datasets.

Protein	% Predicted Disordered X-ray^a^	% Predicted Disordered NMR	% Predicted Disordered
PDBS90	24 ± 2 (14 ± 2)	34 ± 2 (32 ± 1)	24 ± 2 (15 ± 1)
HIV	27 ± 2 (16 ± 1)	50 ± 3 (41 ± 3)	34 ± 2 (19 ± 2)
Influenza	21 ± 2 (10 ± 2)	34 ± 3 (40 ± 3)	21 ± 2 (11 ± 2)

^a^Percentage of residues that are predicted to be disordered that have been characterized by X-ray crystallography.

Table presents means of predicted disorder by PONDR^® ^VLXT. The corresponding PONDR^® ^VL3 are shown in brackets. The total number of proteins used in this analysis was 34 for lentivirus, while the number of influenza A analyzed proteins is 27.

### Averaged predicted disorder rates enable analysis of the viral protein disorder

Table 2 provides a simple measure to classify a given protein as ordered or disordered by prediction. For example, the averaged predicted disorder rate for proteins from PDBS90 is 24 ± 2 (15 ± 2). If this value is used as a benchmark for labelling a protein as moderately disordered or mostly structured, then any protein that falls close to this number with respect to percentage of predicted disordered residues can safely be classified as 'moderately disordered' by prediction. The information in Table 2 provides the benchmarks for the analysis of the results shown in Tables 3 and 4. Table 3 categorizes proteins found in the HIV virus by their function and arranges data by protein location in the virion. The envelope proteins are placed on the forefront with gp120 being the Surface Unit (SU). Just below the SU lies the gp41, which is a transmembrane protein (TM). Using the information in Table 2, we can tell that gp120 can be considered as quite ordered by prediction, whereas the gp41 should be categorized as rather disordered. The amount of predicted intrinsic disorder is high in matrix and capsid proteins, as well as in Vpr and Tat proteins. Nef protein and integrase are predicted to be moderately disordered, whereas both protease and reverse transcriptase contain the least amount of predicted disorder.

**Table 3:** Summary of the predicted disorder rates in HIV proteins.

Protein	Accession numbers	%VLXT(VL3)^a^	Methods^b^	Virus Type
SU	2i60.pdb, Subunit G	11 (0)	X-Ray	HIV-1
(gp120)				
TM	2cmr.pdb, Subunit: S	34 (27)	X-Ray	HIV-1
(gp41) TM	1jek.pdb, Subunit: A	64 (0)	X-Ray	Visna
MA (p17)	1hiw.pdb, Subunit: A	61 (48)	X-Ray	HIV-1
VPR	1ceu.pdb, Subunit: A	39 (63)	NMR	HIV-1
VPU^c^	2goh.pdb, Subunit: A	26 (NA)^d^	NMR	HIV-1
CA (p24)	1afv.pdb, Subunit:A	48 (0)	X-Ray	HIV-1
PR (p11)^e^	1tcw.pdb, Subunit A	18 (0)	X-Ray	SIV
RT (p66)^e^	2hnz.pdb, Sunbunt: A	18 (10)	X-Ray	HIV-1
IN (p32)^e^	1k6y.pdb, Subunit: A	26 (7)	X-Ray	HIV-1
Nef^c^	1avz.pdb, Subunit: A	26 (31)	X-Ray	HIV-1
Tat^c^	1jfw.pdb, Subunit: A	100 (100)	NMR	HIV-1

^a^Percentage of residues in a chain that are predicted to be disordered.

^b^Methods used to characterize the proteins

^c^Locations of these proteins in virions have yet been determined. VPU is however, placed alongside with VPR for comparative purposes only.

^d^Chain available is too short for VL3 analysis.

^e^Exact locations of these proteins have yet been determined.

**Table 4:** Summary of the predicted disorder rates in influenza viral protein.

Protein	Accessions	%VLXT(VL3)^a^	Method
HA1 (H1)	1ruz.pdb, Subunit H	12 (0)	X-ray
HA2 (H1)	1ruz.pdb, Subunit:I	12 (0)	
HA1 (H3)	1mqn.pdb, Subunit: A	25 (0)	X-ray
HA2 (H3)	1mqn.pdb Subunit: B	19 (2)	
NA (N1)	2hu0.pdb,/Sunit: S	8 (0)	X-ray
M1	1ea3.pdb, Subunit: A	25 (0)	X-ray
NS1	1xeq.pdb, Subunit: A	69 (63)	X-ray
NS2	1pd3.pdb, Subunit: A	60 (23)	X-ray
PB1	2hn8.pdb, Subunit: A	47 (NA)	NMR
NP	2iqh.pdb, Subunit: A	44 (0)	X-ray

^a^The percentage of residues that are predicted ordered by VLXT. The parenthesized values are those in VL3.

Table 4 gives the data on the abundance of disorder in influenza A virus proteins. These data provide comparisons of proteins from orthomyxovirus and lentivirus. The envelope of the orthomyxovirus contains hemagglutinin (HA) and neuraminidase (NA) protein. While the percentage predicted disorder rate for gp120 does not vary much by strains, predicted intrinsic disorder in both HA and NA vary significantly by subtype (see Table 5). The M1 is a matrix protein, which provides a link between the surface protein and the capsid. M1 is predicted to be moderately disordered. Both non-structural proteins of the influenza A viruses were predicted to be rather disordered. Similarly, both nucleoprotein and main core protein were predicted to contain significant percentage of disordered residues.

**Table 5:** Predicted intrinsic disorder in surface proteins of influenza virus.

Disorder prediction in various NA subtypes				
Protein	Accessions		%VLXT(VL3)	Method

N1	2hu0.pdb, Subunit A		8 (0)	X-Ray
N2	2f10.pdb, Subunit: A		25 (13)	X-Ray
N4	2htw.pdb, Subunit A		4 (0)	X-Ray
N6	2w20.pdb, Subunit: A		15 (27)	X-Ray
N8	2htr.pdb, Subunit: A		4 (48)	X-Ray
N9	1jsn.pdb, Subunit S		15	X-Ray

Disorder predictions in various HA subtypes				

Protein	Subunit	Accessions	%VLXT(VL3)	

H1	HA1	1ruz.pdb, Subunit H	12 (0)	X-Ray
	HA2	1ruz.pdb, Subunit:I	12 (0)	
H3	HA1	1mqn.pdb, Subunit: A	25 (0)	X-Ray
	HA2	1mqn.pdb, Subunit: B	19 (1)	
H5	HA1	2ibx.pdb, Subunit: A	12 (2)	X-RAY
	HA2	2ibx.pdb Subunit: B	13 (0)	
H7	HA1	1ti8.pdb, Subunit: A	22 (11)	X-Ray
	HA2	1ti8.Pdb, Subunit:: B	30 (0)	

Table 6 summarizes some trends in the distribution of intrinsic disorder among various functional classes of proteins derived from the analysis of literature. First, a collection of RNA-binding proteins show a strong tendency to be highly disordered, both experimentally and computationally. The next sets of proteins that have also been observed disordered, though not as highly disordered as RNA-binding proteins, are DNA-binding proteins. Single-span membrane proteins also contain significant intrinsic disorder, except for the segment that crosses the membrane, which is typically predicted to be ordered. Finally, various enzymes as well as transmembrane proteins (e.g. pores) are among the polypeptides with the least intrinsic disorder. Overall, the results in this table are consistent with previous studies on all of the SwissProt proteins, which were partitioned by functional annotation [24-26].

**Table 6:** Summary of protein types generally predicted to be ordered or disordered. Information is deduced from the literature data mainly reported in [23-26].

Protein	Observed/Predicted Disorder
RNA Binding Proteins	Highly Disordered
DNA Binding Protein	Disordered
Enzymes (e.g. Proteases, Ribonucleases)	Ordered
Non-Multiply Spanning Membrane Proteins	Disordered
Transmembrane Proteins (e.g Pores)	Ordered

**Figure 2:**
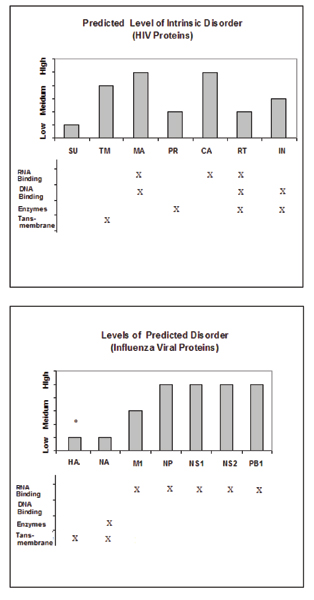
**Chart summarizing the percentages of residues predicted to be disordered in HIV-1 (A) and the influenza A virus proteins (B)**. Top of each panel represents chart of proteins with percentages of residues that are predicted to be disordered by PONDR^® ^VLXT. The types of proteins are also summarized. It should be noted that the percentages of predicted to be disordered residues of HA vary with subtypes, but HA is essentially predicted to be quite ordered. More information is available in Table 5.

Figure 2 presents data for the disorder distribution within the major proteins from HIV and influenza viruses. The proteins are labeled as "predicted to be ordered," "moderately disordered," or "very disordered." The qualitative values are assigned according to the PONDR^® ^VLXT values by comparison to the mean values of Table 2. Here, the proteins whose percentages of predicted disordered residues are between 30–40% were considered as moderately disordered. The proteins with the disorder scores above 40% and below 20% were considered as very disordered or as ordered, respectively. Figure 2 shows that the amount of predicted intrinsic disorder increases as the protein becomes located closer to the viral core, which is the site of the genomic RNA. In general, a comparison of Tables 3, 4 and Figure 2 with the Table 6 indicates that viral proteins follow the trend in the distribution of intrinsic disorder among functional classes previously described for other proteins.

## Discussion

### Predicted disorder varies with protein type, protein location and function

#### Predicted disorder varies with location of protein in virion

There is an interesting correlation between the percentage disorder rate and the protein localization within the virion. This phenomenon is especially clear for influenza virus (see Figure 2), where the closer the protein gets to the core, then higher the level of predicted disorder. This can easily be explained by the increased likelihood of colocalization of the RNA-binding proteins and the genomic RNA in the viral core. This trend is also seen for HIV proteins with the exception for the several enzymes, which are located in the close proximity to the core (see Figure 2). But of course enzymes need to be structured so the active site can provide a catalytic surface, and so this result is entirely consistent with previous work [24-26].

#### Hemagglutinin versus gp120

Data in Tables 3 – 4 and Figure 2 show that the surface proteins are generally predicted to be ordered in both the influenza and HIV viruses. HA is crucial in the entry of the virus to the host. A cleavage at the disulfide bond between subunits, HA1 and HA2, has to occur before the viral entry can take place. The function of HA can be compared to that of gp120, as gp120 is also known to play a crucial role in mediating the entry of the virus into the host cell [1,27]. Neuraminidases, on the other hand, play enzymatic role at the other end of the viral process. This protein cleaves the sialic acid group from the oligosaccharide portion. This cleavage step is needed for the virion to be released into the extracellular region [28].

According to our analysis, gp120, like HA, is predicted to be quite ordered. There are however, observed differences in the prediction results for these two proteins: gp120 was consistently predicted to be ordered, whereas the levels of predicted order in influenza HA varied with the viral subtype. A summary of this can be found in Table 5. It is also should be noted that both HA (HA2) and NA are transmembrane proteins [28,29].

#### Transmembrane proteins are more ordered

In HIV, a protein that spans the lipid membrane is the transmembrane (TM) protein, gp41. This protein acts as a fusion protein [30] and functions in membrane interactions. This integral membrane protein contains a TM anchor domain that holds this envelope protein in association with the lipid bilayer [31]. This TM protein is responsible for the fusion of the viral and cellular membranes via its fusion peptide located in its extracellular, N-terminal domain [32]. Previously transmembrane fragments of channels and pores were predicted to be highly ordered [24-26]. However, the situation might be quite different for membrane proteins with relatively large extra- and intracellular domains, e.g., for fusion proteins. We now have an opportunity to analyse the predicted disorder rate of transmembrane proteins that are involved in the membrane fusion. Table 3 shows that the predicted disorder level for gp41 is quite sizeable (34% for PONDR^® ^VLXT). In fact, the amount of disorder in this protein is significantly higher than that of the transmembrane proteins (HA, NA) of the influenza A virus. This might be also correlated with the high level of predicted disorder in the HIV matrix.

Similarly to gp41, HA is a transmembrane glycoprotein. Both, gp41 and HA, are members of the class I viral fusion proteins that mediate viral entry into cells. Class I viral fusion proteins are thought to fold into a prefusion, metastable conformation, which is then activated to undergo a large conformational rearrangement to a lower energy state, thereby providing the energy needed to accomplish membrane fusion [33-35]. The role of HA as a fusion protein and the associated large-scale conformational changes may help to account for the slightly higher predicted disorder rates observed for HA in many subtypes (see Table 5).

Analysis of the data in Table 3 revealed that transmembrane viral proteins are, in general, characterized by relatively low predicted disorder rates. For instance, the Vpr protein which is present in HIV but is not expressed in SIV [36] and structure of which was determined by NMR is predicted to be rather disordered (39% by PONDR^® ^VLXT and 64% by PONDR^® ^VL3, Table 3). On the other hand, Vpu was predicted to be more ordered (26% by PONDR^® ^VLXT) in agreement with the fact that this protein has a transmembrane domain [37].

#### More disorder at the core

The matrix proteins, which form a layer below the lipid envelope, produced interesting data for both families of viruses. The matrices of both influenza and HIV viruses are relatively disordered (see Tables 3 and 4). The HIV matrix protein is predicted to be highly disordered, whereas the influenza virus matrix protein is predicted to be only moderately disordered (or somewhat ordered) by PONDR^® ^VLXT. This peculiarity may highlight an important difference between the two families of viruses and may have important medical implications. For the influenza virus, the proteins that are even closer to the core include NS1, NP and PB1. All these proteins are predicted to be highly disordered.

#### More disorder at the core unless proteins are enzymes

While M1 is known to bind RNA, proteins that are located closer to the core are even more likely to interact with the viral RNA. This may account for the trend that, for proteins that are closer to the core, the amount of predicted disorder increases. This trend is clearly highlighted in Figure 2. Interestingly, a number of RNA-binding proteins that are not viral have also been predicted to be disordered (Table 6). This trend can also be seen for HIV virus proteins, with the exception that enzymes are usually predicted to be ordered. Intergrases (IN), Reverse Transcriptase (RT), and to some extent Protease (PR) exemplify the observation that enzymes are exceptions to the general trend. That is, all three of these proteins are enzymes, and they are all predicted to be relatively ordered. Reverse transcriptase and integrases, however, are also RNA-binding proteins, with the RT having an additional ability to bind DNA. In such cases, the proteins typically have (nucleic binding) regions that are somewhat disordered and also (catalytic) regions that are highly ordered respectively. Such trends have been observed for non-viral proteins in general [24-26], and the viral proteins seem to follow this rule of thumb as well. Consistent with these trends, the HIV protease, which binds neither RNA nor DNA, is not surprisingly predicted to be the most ordered of all.

### Predicted disorder in hemagglutinin might correlate with viral infectivity

#### Loss of infectivity for specific virus subtype via fatty acid deprivation

The attachment and membrane fusion of the influenza virus and the host cell are mediated by its hemagglutinin (HA). HA is a homotrimer, and each monomer comprises an ectodomain with about 510 amino acid residues, a transmembrane domain with 27 residues, and a cytoplasmic domain with 10 to 11 residues. The HA monomer is synthesized as a single polypeptide chain and cleaved into two subunits, HA1 and HA2, by proteolytic enzymes after virus budding or during intracellular transport. The HA1 and HA2 subunits are functionally specialized. HA1 carries receptor-binding activity, and HA2 mediates membrane fusion [38]. As discussed briefly above, the amount of intrinsic disorder in HA1 and HA2 varies with the viral subtype (Table 5). Analysis of the past experimental data in comparison with the disorder predictions in Table 5 suggests that variations in the infectivity of the virus [39], variations in the assembly of the HA proteins, and variations in the correlation between protein-membrane interaction and the lipid raft motion may all be related to the amount of predicted disorder. For example, one of the HA functions is to assemble proteins, including those involved into the formation of pores. Acetylation of the HA molecules often affects this function, which is crucial for the infectivity of the virus. However, it has been shown that H1, H3, and H7 behave differently when the sites that are normally palmitylated are mutated. Viral subtypes with H1 proteins were most affected by the mutations, whereas the virions did not lose much of their infectivity in the case of H3 and H7 [40,41]. Table 5 shows that, among the viral subtypes analyzed, H1 proteins possess the least amount of predicted disorder, whereas H3 and H7 proteins were predicted to be essentially more disordered. We showed elsewhere that enzyme-mediated posttranslational modifications usually occur with disordered regions [24-26,42]. The increased predictions of intrinsic disorder in HA are associated with increased infectivity of influenza virus, perhaps via changes in posttranslational modification the ease of which may depend on the tendency to be disordered.

#### Intrinsic disorder may provide a bypass to the lipid raft requirement

For all enveloped viruses, the envelope is derived from the host cell during the process of virus budding. In the case of influenza virus, budding takes place at the apical plasma membrane and is heavily dependent on the presence of lipid microdomains, or "rafts" [43-45]. Lipid rafts, also known as detergent-insoluble glycosphingolipid-enriched domains, are specific domains on plasma membranes that are enriched in detergent-insoluble glycolipids (DIGs), cholesterol and sphingolipids [46-48]. Levels of cholesterol and sphingolipids can vary amongst individuals, which alters the extent the raft formation. Lipid rafts play an important role in several biological processes, including signal transduction, T-cell activation, protein sorting, and virus assembly and budding [48]. Such enveloped viruses incorporate some integral membrane proteins; among the best studied are the influenza virus hemagglutinin (HA) and neuraminidase (NA) [49]. Acetylation of the envelope proteins and also palmitoylation are important for these viral proteins to be targeted to the lipid raft microdomains on the cell surface [50]. C-terminal domains of both HA and NA of influenza virus are crucial for association with rafts and this interaction constitutes part of the signaling machinery necessary for apical targeting in polarized cells. In fact, the cytoplasmic tails of HA and NA are so important for assembly that the information contained in these tails is partially redundant [51]. For example, the removal of the cytoplasmic tail or mutation of the three palmitoylated cysteine residues in the transmembrane (TM) domain and the cytoplasmic tail of influenza virus hemagglutinin (HA) was shown to decrease the association of HA with lipid rafts, decrease the incorporation of HA into virions [44], and modulates incorporation of cholesterol into the viral envelope.

The level of the envelope cholesterol has been shown to play a crucial role in the HA-mediated fusion of the influenza virus with the host cell [52]. These data were obtained for the WSN (H1N1) strain of influenza virus and the authors proposed that differences may exist with other virus strains. Perhaps the virion cholesterol is important for the organization of influenza virus HA trimers into fusion-competent domains, and perhaps also the depletion of cholesterol inhibits virus infectivity due to inefficient fusion [52]. Here we suggest that variations in intrinsic disorder in the surface proteins may play similar role. In fact, Table 5 shows that H1 is predicted to be ordered, whereas H3 and H7 are predicted to be more disordered. This increased level of disorder might offer a mechanism for proteins to by-pass the lipid raft requirement. Studies on chimera proteins with specific swapping of regions predicted to be ordered or disordered could be used to test this proposed mechanism.

### Disorder or order pairing of HA and NA may be intertwined with the evolution of the influenza viruses

#### Ordered-ordered versus disordered-disordered HA and NA in influenza A virus serotypes

As has already been mentioned, sixteen HA serotypes and nine NA subtypes of influenza A virus are known. Among the three influenza types, the type A viruses are the most virulent human pathogens that cause the most severe disease. The list of some influenza A virus serotypes with the largest known human pandemic deaths includes H1N1 ("Spanish flue"), H2N2 ("Asian flue"), H3N2 ("Hong Kong flu"), and H5N1 ("Avian flue"). Table 7 illustrates an interesting correlation between the amounts of predicted intrinsic disorder in HA and NA proteins from the different influenza A virus serotypes: in H1N1 and H5N1 subtypes, both HA and NA are predicted to be ordered, whereas H3N2 serotype is characterized by more disordered hemagglutinin and neuraminidase. Perhaps such a combination is not coincidental but is instead evolutionarily preferred.

**Table 7:** Observed paring of predicted disorder of HA-NA in subtypes involved in major epidemics. Quantitative details of the respective predicted disorder values can be found in Table 5.

Subtype	Description	HA (Predicted Disorder/Order)	NA (Predicted Disorder/Order)
H1N1	"Spanish Flu" (1918)	Ordered	Ordered
H2N2	"Asian Flu" (1957)	NA^a^	More Disordered
H3N2	"Hong Kong Flu" (1968)	More Disordered	More Disordered
H5N1	"Avian Flu" (1997)	Ordered	Ordered

^a^PDB structure of H2 has not been found, presumably, because the H2N2 subtype evolved quickly into H3N2.

### Disorder as a viral weapon for evading the immune response

An understanding of viral surface proteins is crucial for developing the appropriate vaccination strategies and for improving the understanding of the immune responses. The comparative analysis of intrinsic disorder distribution in the HIV and influenza virions uncovers specific patterns that could provide some useful insight into these problems. Above we showed that the level of predicted disorder varies in the HA and NA subtypes. This observation might be used for tuning vaccination strategies. However, the data in Table 5 shows that the variations in the predicted disorder do not deviate greatly. Furthermore, in general, HA and NA can be described as highly ordered to or moderately disordered (see Tables 3 and 5, and Figure 2). This may account for the observations that the anti-influenza antibodies recognize and bind various HA and NA subtypes providing the grounds for the development of an effective immune response and therefore for the elaboration of the appropriate vaccination strategies. The situation with immunogeneity of HIV virus is totally different. Although antibodies were found to bind to several HIV proteins, these HIV-binding antibodies do not lead to an effective immune response. The reason for this is unknown as of yet. However, we believe that a comparative analysis of disorder distribution in proteins from both orthomyxoviruses and lentiviruses might potentially provide greater insight into this problem.

The first step in HIV infection is the binding of the envelope glycoprotein gp120 to the host cell receptor CD4 [53,54]. CD4 binding induces extensive structural rearrangements in gp120, resulting in the exposure of a binding surface for the second host cell chemokine receptor, CCR5 or CXCR4 [55,56]. The interface between gp120 and CD4 is highly conserved among different HIV-1 isolates [57]. In gp120-CD4 complexes, CD4 was shown to interact with all three domains of gp120, including the inner domain, the outer domain, and the bridging β-sheet. Furthermore, in all structures of various gp120-CD4 complexes analyzed by X-ray crystallography, a deep hydrophobic cavity enclosed by conserved gp120 residues was detected [58]. CD4 residue Phe43 is the only cavity-interacting residue in CD4. It fits to the opening of this cavity [57] and was shown to contribute about 23% of the total interaction surface [58]. According to our analysis, the surface protein of the HIV virion, gp120, has a consistently low predicted disorder value across various strains of lentiviruses (data not shown). Therefore, this feature has functional implications since a rigid structure of gp120 might be necessary for the formation of a stable complex with the host protein, CD4.

The analysis of Figure 2 reveals that, unlike the proteins of influenza A virus, HIV proteins do not follow the trend of having increasing amount of predicted disorder as the locations of the proteins become closer to the core. In part, this distinct trend can be attributed to the presence of several enzymes in the HIV virion capsule. However, even the HIV matrix proteins and some of the surface proteins have quite high percentages of residues predicted to be disordered. For example, Tables 3 and 4 shows that the matrix protein of influenza virus (M1) is predicted to be only somewhat disordered (25% by PONDR^® ^VLXT and 0% by PONDR^® ^VL3), whereas the matrix protein of HIV, MA (p17), has a very high percentage of disordered residues (61% PONDR^® ^VLXT and 48% by PONDR^® ^VL3). Furthermore, Table 3 shows also that this very high abundance of intrinsic disorder is extended to areas above the matrix and is observed in the HIV envelope. The gp41 (TM), which is a transmembrane protein involved in fusion, has a high predicted disorder rate, in striking contrast to the fusion protein of the influenza virus (HA2, Table 5).

An interesting possibility is that the high prevalence of intrinsic disorder in proteins located in the close proximity to the surface of HIV- related viruses provides a mechanism for the avoiding the induction of immune response. In fact, the antigenicity of a given protein is known to reside in a restricted number of antigenic determinants (sites or epitopes) located on its surface. As antigenic determinants of several proteins have been shown to correspond to the surface regions with high segmental mobility (high B-factor values), the high mobility of an antigenic determinant was suggested to help in the determinant adjustment to a pre-existing antibody site not fashioned to fit the exact geometry of a protein [59]. On the other hand, additional research has revealed that an effective antigenic site, being mobile, should possess an internal propensity to form ordered structure; i.e., it should not be completely disordered. Importantly, some long disordered regions and intrinsically disordered proteins promote weak immune responses or are even completely non-immunogenic [60-62]. This is further illustrated by the analysis of literature data on the gp120 immunogeneity.

Neutralizing antibodies play a significant role in the vaccines development. The key HIV targets for neutralizing antibody are found in the external envelope protein, gp120 [63-65]. The principle neutralizing determinant of HIV-1 virus was mapped to the third variable (V3) loop region (residues 301–341) of gp120 [66-68]. This V3 loop is also required for viral entry into target T cells and macrophages [69] and interacts with chemokine co-receptors on the surfaces of these cells [55,56,70]. The V3 loop is characterized by a highly variable amino acid sequence, which is assumed to contribute to the ability of HIV to escape the host immune response [71].

Using solid state NMR spectroscopy it has been shown that a 24-residue fragment of the V3 loop of HIV-1 strain III (namely residues 308–331) that includes the GPGR motif is conformationally heterogeneous [71]. Furthermore, this fragment was shown to adopt very different conformations when bound to different anti-V3 antibodies [71-73]. The disorder-to-order transition of V3 loop has been hypothesized to play a crucial role in function of this protein, determining its potential to interact with a variety of chemokine receptors and thus allowing different avenues into the cell [71]. The same mechanism makes devising vaccines against HIV very difficult because some V3 loops escape detection by antibodies that specifically recognize a particular conformation but that fail to bind other conformations. These observations provide further support to the hypothesis that high abundance of intrinsic disorder in proteins located in the close proximity to the surface of HIV-1 can help this virus to avoid the immune response induction. Therefore, intrinsic disorder might represent a crucial viral weapon for evading immune response.

We previously discussed several pathogens that use disordered regions for binding, with these disordered regions being weakly immunogenic, and suggested using disorder for binding might be a common strategy for avoiding the immune system [60]. For this mechanism, the disordered region needs to have a sufficiently high flexibility. For such flexible disordered regions, we speculate that the relatively small size of the antibody binding site provides insufficient binding energy to fold the flexible disorder and therefore cannot bind tightly enough for the generation of an immune response. On the other hand, in our proposal the relatively larger size of the receptor binding surface can provide sufficient energy of association to overcome the flexibility and thereby induce binding via a disorder-to-order transition. The flexibility of the key HIV proteins may be slightly less than that for the previously discussed pathogens, and so antibodies are produced and these bind to different conformational states. Yet the ability of the flexible disordered binding region to fold in different ways may lead to confusion of the immune system and may substantially weaken the overall immune response as discussed above. Thus, antigenic sites may benefit by being somewhat flexible [59], but probably become less effective as the flexibility increases beyond some useful level.

## Conclusion

Results presented in this paper show the usefulness of the intrinsic disorder prediction for the comparative analysis of viral proteins. This approach offers several advantages, including the opportunity to map proteins by functionality, predicted disorder, and locality across viral species, strains and subtypes. Furthermore, it provides useful benchmarks for the evaluation of the intrinsic disorder concept and for the analysis of various disorder predictors. Using this comparative study of predicted disorder, several interesting patterns in the behaviour of viral proteins from HIV-1 and influenza A viruses were uncovered. We have shown that the patterns of predicted disorder can be mapped and related to the functions of the various proteins. There is evidence that the functions and the amount of disorder of the proteins are related to their physical location in the virion. Some of the key findings of this paper are further outlined below.

Intrinsic disorder is unevenly distributed within the virions, especially for influenza, with the least predicted disorder being observed at the surface proteins and the most disorder being characteristic for the proteins at the virion core. While a similar trend is observed for HIV, the disorder changes are much less pronounced.

Proteins near the surface of HIV-related viruses are characterized by higher levels of predicted disorder as compared to influenza. Although the major surface protein, gp120, has been consistently predicted to be ordered, its major neutralizing determinant is highly mobile. These data support a scenario where HIV virions can escape immune response despite the availability of antibodies for the HIV-related proteins.

Significant variations in the amount of predicted disorder by HA subtypes in influenza A virus were observed. This might provide an explanation for the variations in the functionality and infectivity of specific viral subtypes. Furthermore, NA and HA of major influenza A pandemic, tend to pair in such a way that both tend to be predicted either ordered-ordered or disordered-disordered. Such behaviour might be linked to the evolutionary advantages of being ordered or disordered, but more experiments are needed to test this conjecture.

## Methods

### Tools and materials used

The programs were written in C#, JAVA^®^-JDBC, Microsoft^® ^SQLSERVER, and MYSQL. Object-oriented programming in JAVA^®^-5 was also used. The design of the database was done using relational database concepts with normalization in Third Normal Form Boyce-Codd Normal Form [74].

### PONDR^® ^VLXT and VL3 predictors

The predictors of intrinsic disorder used in this paper are PONDR^® ^VLXT and VL3 [18,19,21]. PONDR^® ^VLXT was built using 15 proteins whose structures were elucidated using X-ray diffraction, NMR spectroscopy, circular dichroism spectroscopy, or limited proteolysis [19]. PONDR^® ^VL3, on the other hand, was built using a combination of 30 neural networks and a training set of disordered regions of 150 proteins [21].

### Relational database and entity relationship diagram

In order to do a comparative study of the viral proteins, it was necessary to develop a database that would capture the information from the amino acid sequence and the disorder prediction. The list of proteins of interest included viral proteins of lentiviruses and orthomyxoviruses. Searches were done on the list using the Entrez website [22]. Available samples were randomly chosen with preferences given to those with longer chains and those with binding partners. Whenever possible, corresponding viral protein of different virus strains were included as samples and annotated. The respective FASTA and PDB [22] files were downloaded and stored using a JAVA^® ^program and the list prepared.

In order to provide a benchmark for predicted disorder, a set of proteins from PDB-Select 90 was randomly chosen and downloaded to the database. The mean and standard deviation were calculated using bootstrapping techniques when necessary [75]. PDB Select90 [23] is defined as a representative, non-redundant subset of the PDB [22], made up of proteins that have no more than 95% sequence identity [23].

**Figure 3:**
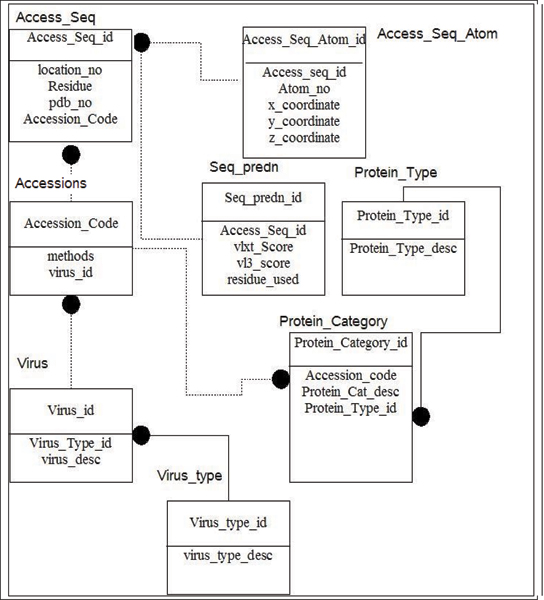
**An entity-relationship (ER) diagram of the viral protein database**. Each entity contains attributes. The type of relationships is shown by the connector and/or the black circle. For example, a black circle followed by dotted line indicates a many to zero or one relationship. Whereas, the black circle followed by a solid line indicates a many to one relationship [76].

Figure 3 shows the Entity-Relationship (ER) Diagram [76]. The ER Diagram attempts to summarize the relationships among entities in a relational database. In our case, entity Virus_Type contains information about the virus family (such as Lentiviradae), while entity Viruses contains names of viruses such as HIV-1 on the virus_id attribute. In order to query by protein types, the entities protein_category and protein_type were designed. Attributes for protein category includes accession_code and protein_cat_desc, which include descriptions of proteins such as p17. Protein_type_id links the Protein_category entity to the Protein_Type entity, which contains information relayed to protein type, e.g. matrix protein.

Using a set of programs written in JAVA^®^, the PDB and FASTA files were searched, and the essential information was placed in the MYSQL tables using accessions seq_access and seq_access_atom. The necessary FASTA files were then used to generate PONDR^® ^VLXT and PONDR^® ^VL3 scores via a LINUX BASH shell script. Another JAVA^® ^program was then used to load the prediction into the seq_predn table. Information regarding to the virus and its subtype was initially stored in a Microsoft^® ^SQLSERVER database via C# and later was transferred to the MySQL database server.

## Competing interests

The authors declare that they have no competing interests.

## Authors' contributions

GG has designed and implemented the experiments. AKD and VNU have provided advice and participated in the manuscript writing.
